# Negative pressure wound therapy use to decrease surgical nosocomial events in colorectal resections (NEPTUNE): study protocol for a randomized controlled trial

**DOI:** 10.1186/s13063-015-0817-8

**Published:** 2015-07-30

**Authors:** Sami A. Chadi, Kelly N. Vogt, Sarah Knowles, Patrick B. Murphy, Julie Ann Van Koughnett, Muriel Brackstone, Michael C. Ott

**Affiliations:** Division of General Surgery, Department of Surgery, University of Western Ontario, London, ON Canada; Department of Surgery and Surgical Oncology, Schulich School of Medicine & Dentistry, Western University, LHSC, Victoria Campus, 800 Commissioners Rd E, London, ON N6A 5W9 Canada

**Keywords:** Surgical site infection, Colorectal resection, Negative pressure wound therapy, Prevena™

## Abstract

**Background:**

Surgical site infections (SSIs) are the second most common form of nosocomial infection. Colorectal resections have high rates of SSIs secondary to the inherently contaminated intraluminal environment. Negative pressure wound therapy dressings have been used on primarily closed incisions to reduce surgical site infections in other surgical disciplines. No randomized control trials exist to support the use of negative pressure wound therapy following elective open colorectal resection to reduce surgical site infection.

**Methods/Design:**

In this single-center, superiority designed prospective randomized open blinded endpoint controlled trial, patients scheduled for a colorectal resection via a laparotomy will be considered eligible. Patients undergoing laparoscopic resection will be enrolled but only randomized and included if the operation is converted to an open procedure. Exclusion criteria are patients receiving an abdominoperineal resection or a palliative procedure, as well as pregnant patients and those with an adhesive allergy. After informed consent, 300 patients will be randomized to the use of a standard adhesive gauze dressing or to a negative pressure wound device. Patients will be followed in hospital and reassessed on post-operative day 30. The primary outcome measure is SSI within the first 30 post-operative days. Secondary outcomes include the length of hospital stay, the number of return visits related to a potential or actual SSI, cost, and the need for homecare. The primary endpoint analysis follows the intention-to-treat principle.

**Discussion:**

NEPTUNE is the first randomized controlled trial to investigate the role of incisional negative pressure wound therapy in decreasing the rates of surgical site infections in the abdominal incisions of patients following an elective, open colorectal resection. This low-risk intervention may help decrease the morbidity and costs associated with the development of an SSI in our patients.

**Trial registration:**

NCT02007018 – clinicaltrials.gov; 5 December 2013

**Electronic supplementary material:**

The online version of this article (doi:10.1186/s13063-015-0817-8) contains supplementary material, which is available to authorized users.

## Background

Surgical site infections (SSIs) are the second most common type of nosocomial infection and represent a significant burden on the healthcare system [1, 2]. The morbidity, increased length of stay, delay in further treatment (adjuvant chemotherapy or radiation) as well as the significant psychological effects on patients has been well demonstrated in the literature [3, 4]. In 1998, the only cost-analysis related to SSI that was performed in a Canadian center was published. Here, Zoutman and colleagues demonstrated an average of US$4000 in additional costs per patient [5]. Resections of colonic and rectal segments have been associated with the highest rates of SSI amongst cases without perforation of gross intra-abdominal contamination, likely secondary to the inherently contaminated intra-luminal environment [6]. In such circumstances, despite best practice recommendations of pre-operative antibiotic use and aseptic operative technique [4], SSIs are reported at rates of 15 to 30 % [7, 8].

Few interventions beyond pre-operative antibiotic prophylaxis and aseptic technique have been shown to decrease rates of SSI. Negative pressure wound therapy (NPWT) is a relatively new treatment concept, initially reported on in 1997 by Morykwas et al. as a novel therapy for chronic wounds [9].

The first identifiable report in the literature on incisional NPWT (iNPWT) is a case-series from Gomoll et al. in the setting of orthopedic trauma. They demonstrated a decrease in serous exudative rates and good healing of incisions post-operatively [10]. Cardiac surgery patients are known to be susceptible to mediastinitis, associated with significant morbidity and mortality. A retrospective review of 57 such patients who received iNPWT was reported in 2009 whereby an absence of any sternal wound infections was demonstrated [11]. In 2011, Stannard randomized 263 blunt orthopedic trauma patients with high-risk tibial and calcaneal fractures requiring surgical stabilization to either usual care or iNPWT [12]. Here they demonstrated a relative risk of infection of 1.9 times higher in control patients with no significant difference in the injury severity scores of each group. In a recently published series, 27 patients, postabdominoperineal resection, with significant risk factors for wound infection had iNPWT applied to their perineal incisions until the fifth post-operative day [13]. When compared to a recent historical control group, a statistically lower rate of perineal SSI was detected in the intervention group. Furthermore, on multivariate analysis, when other risk factors were controlled for, the use of iNPWT was found to be protective against the outcome of perineal SSI. Finally, Matatov et al. performed a retrospective cohort study using the same NPWT dressing as the current proposal and found a significant difference (24 % versus 6 %) in SSI compared to standard dressing following 115 femoral cut-downs for vascular procedures [14].

Patients undergoing elective, open colorectal surgery are at a high risk of SSI. Given the significant patient morbidity associated with this complication, there is an identifiable need for an intervention to help decrease the rates of SSI in this patient population. Furthermore, there is a paucity of literature investigating the role of iNPWT in colorectal surgery. The objective of this study is to assess the role of iNPWT in patients undergoing an elective, open colorectal resection (CRR) on decreasing the rates of SSI as compared with a standard gauze adhesive dressing. We hypothesize that patients randomized to iNPWT will have a lower incidence of SSI within the first 30 days of surgery compared to patients randomized to the control group. The trial will be a prospective, randomized, superiority trial.

## Methods/Design

A prospective analysis of SSIs at our institution was done to prepare the calculation of power and sample size, and informed the trial protocol and manuscript creation. Our institutional ethics at the University of Western Ontario performed a Full-Board review and approved the trial (REB 104819). The trial is registered at Clinicaltrials.gov (NCT02007018). This protocol conforms to SPIRIT recommendations for the minimum set of scientific, ethical, and administrative elements that should be addressed in a clinical trial protocol (Additional file 1). Financing is granted through an industry grant with Kinetic Concepts Inc. (San Antonio, TX, USA). The study sponsor has no role in data collection, management, analysis, writing or influence on the decision to publish results.

### Study design

This is a single-institution, prospective, randomized, open label, blind endpoint trial design [15] with a superiority framework, in which eligible patients will be randomized to iNPWT or a standard gauze adhesive dressing using block randomization and stratification based on one of two hospital sites.

### Study setting

The study will take place at the two campuses of London Health Sciences Center (LHSC) in London, Ontario, Canada: University Hospital and Victoria Hospital. Both hospitals are academic teaching hospitals and are associated with the Schulich School of Medicine and Dentistry at Western University. Both sites use an enhanced recovery after surgery protocol following colorectal surgery to improve patient outcomes and reduce length of stay [16].

### Eligibility

Patients scheduled for any elective CRR requiring a midline laparotomy are potentially eligible for the study. Patients booked for a laparoscopic approach are eligible to be enrolled but will only be randomized and included if their surgical procedures are converted to open through a midline laparotomy. Eligible surgical procedures include segmental, sub-total or total colectomies as well as low and ultra-low anterior resections. Before enrollment informed consent will be obtained from each patient. Patients will be identified by study personnel and will be enrolled from the pre-operative clinic by a study nurse.

### Inclusion criteria

Patients must be 18 years or older and scheduled for an elective CRR and must undergo an open procedure or laparoscopic procedure converted to open operation. Informed consent must be obtained.

### Exclusion criteria

Patient who undergo abdominoperineal resections and pelvic exenterations, or have a bowel perforation at the time of operation will be excluded. Patients undergoing an operation for palliative purposes (life expectancy less than 3 months), who are pregnant, or who have a known sensitivity/allergy to adhesive material will also be excluded.

### Intervention and control

The intervention addresses the type of dressing applied following an elective, open, colorectal operation. The intervention is iNPWT in the form of the Prevena™ Incision Management System (PIMS) (Kinetic Concepts Inc. (KCI), San Antonio, TX, USA). This is a sponge dressing connected to a hand-held continuous vacuum in a single-use unit. The vacuum is set to 125 mm Hg. The device is applied under sterile conditions in the operating theater and remains on the patient until the earlier of post-operative day 5 or hospital discharge. Any concern about the wound or the device will require removal of the iNPWT earlier than planned, and will be recorded as a secondary endpoint of the trial.

The control dressing is a gauze adhesive dressing which covers the laparotomy incision. The post-operative management is typically removal on day 2 post-operatively with daily dressing changes thereafter. Apart from the dressing applied to the wound in the operating room, and the care of that dressing post-operatively, the patients pre- and post-operative care will be the same in both groups.

As per current best practice in SSI prevention, the following regimen will be applied to all trial patients, regardless of intervention arm. A standardized dose of pre-operative antibiotics (depending on allergy status) directed by the hospital’s local antibiogram will be administered to patients within 30 minutes prior to surgery, and will be re-dosed in procedures longer than 4 hours. Post-operative antibiotics will be allowed for a maximum of 24 hours. Hair will be removed with electric clippers. Skin preparation will be performed with 2 % chlorhexidine unless the patient has an allergy to this preparation.

### Randomization, allocation concealment and blinding

Randomization will take place centrally using random permutated blocks of 4, 6 or 8 and will be stratified based on site (University Hospital or Victoria Hospital) of the operation. After the fascia is closed a member of the surgical team will use a centralized web-server to randomize the patient. The surgical team, clinical staff, and patient will not be blinded to the intervention status. The primary outcome of SSI will, however, be blinded to the outcome assessors, as the dressing (iNPWT or adhesive gauze) will be removed by the clinical team prior to the first visit from the outcome assessors on post-operative day 5 or the day of hospital discharge. The outcome assessors are blinded and will assess the wound daily after the dressing is removed until hospital discharge. A minority of wounds are expected to be seen by the on-call team and it is unlikely the resident or physician involved will know the patient’s treatment allocation.

### Definition of endpoints and outcome measures

The primary endpoint is the incidence of SSI within 30 days of surgery. SSI is defined by the Center for Disease Control and Prevention (CDC) criteria [1] as: infection occurring within the first 30 post-operative days with at least one of the following:

1. Purulent drainage from the incision
2. Organisms isolated from an aseptically obtained culture of fluid or tissue from the incision
3. At least one of the following signs/symptoms of infection:

- Pain or tenderness
- Localized swelling
- Redness
- Heat

In these instances, the incision is deliberately opened by a surgeon (unless the incision is culture negative)

4. Diagnosis of SSI by the surgeon or attending physician.

SSI will be assessed every day following dressing removal, at discharge, 30 days following the operation or at any time during the study period if the patient or surgical team has concerns about the development of an SSI in the laparotomy incision.

### Secondary endpoints

Secondary outcomes assessed will include the need for, and duration of at-home nursing care (home care) related to SSI. The need for home care will be determined by the surgical team after the identification of an SSI. The duration of home care requirements will be determined at the time of post-operative clinic visits, and by contact with the home care provider. Additional secondary outcomes assessed will include the length of hospital stay, the number of return visits related to a potential or actual SSI, and cost. The cost will be determined based on the average cost provided by LHSC for inpatient, emergency department (ED) or clinic care, and billing fees for both the surgeon and anesthetist for any return trip to the operating room. Standardized time and materials costs will be obtained using available data from the Community Care Access Center for home care. A return visit related to an SSI will include any visit to the ED, outpatient surgical clinic, or requirement for admission to hospital that is deemed by the primary surgeon or study team to be due to a) signs or symptoms of SSI requiring assessment; b) development of an SSI requiring management; or c) ongoing care of a known SSI. The management cost of an SSI will be calculated for each instance. Outcomes related to potential harm will also be identified including local reaction to the iNPWT device, and the need for early removal of the dressing.

### Sample size

Our pilot study revealed an SSI rate of 35 % in patients undergoing colorectal surgery at our institution. Based on previous studies and the clinical experience of the study’s senior investigators, a reduction in the rate of SSI to 20 % (absolute risk reduction 15 %; relative risk reduction 43 %) may be expected. This is the minimum clinically relevant reduction both from a patient outcome perspective and a cost reduction standpoint. Given an alpha of 0.05, for 80 % power, this yields a required sample size of 138 patients per group. The planned sample size will be inflated for potential loss of follow-up, and will be rounded for a final sample size of 150 patients per group and a total study sample of 300 patients. Loss to follow-up is felt to be a minimal concern as patients are required to attend their 30-day post-operative visit to maintain the therapeutic relationship with the surgeon for oncological and disease management.

### Data collection and management

All data collected (baseline characteristics, primary and secondary outcomes) will be recorded through an online data collection portal and be maintained behind a secure server and firewall. Data will be collected by the study nurse, trial personnel and surgical teams. A Data Safety and Monitoring Board (DSMB) will be assembled consisting of three surgeons not involved in patient accrual or in study administration. Given previous demonstration of the safety of iNPWT, we expect minimal to no harm. Accuracy of data collection through the online secure data collection forms will be ensured by the DSMB by performing sample assessments at regular intervals. The severity of adverse events will be evaluated using the Common Terminology Criteria for Adverse Events (CTCAE) v4.0 grading scale (see http://ctep.cancer.gov). Any grade 4 or grade 5 events will be reported to the primary investigator, the DSMB, and the institutional research ethics board as well as the device company headquarters through established reporting systems.

### Statistical analysis

All analysis will be pre-specified and conducted according to the intention-to-treat principle with the use of SPSS version 20 (SPSS Inc., Chicago, IL, USA). The proportion of SSIs amongst all patients undergoing CRR will be compared between the iNPWT and adhesive gauze arms using a chi-square test. An absolute risk increase/reduction for SSI will be presented for the use of iNPWT, as well as the number needed to treat to prevent a single SSI. Data will be analyzed according to the intention-to-treat principle. Patients lost to follow-up will be considered to have developed an SSI for the purposes of primary analysis. Furthermore, despite instructions to contact or return to clinic if any concerns of an SSI arise, any incisions diagnosed or treated for an SSI by any physician will be considered as having had an SSI.

Baseline characteristics of the two groups will be recorded, including body mass index (BMI), self-reported smoking status and co-morbidities. In the event that any of the baseline characteristics are found to differ significantly between the intervention and standard care groups by chance, secondary analyses will be performed using multivariable logistic regression to adjust for the effect of differences in the baseline characteristics on the primary outcome. Results will then be presented as an adjusted odds ratio.

Secondary outcomes will be compared between groups using a chi-square for categorical variables (i.e., need for home care, proportion receiving antibiotics). Non-normally distributed continuous variables (length of hospital stay, duration of home care treatment, number of return visits) will be compared using the Mann-Whitney *U*-test. As potential harm is expected to be minimal, incidence of allergy/sensitivity to iNPWT will be reported, but no formal statistical analyses are planned. A *p*value of less than 0.05 will be considered significant. The trial will undergo only one set of analyses at trial completion. No interim monitoring is planned as, given the small sample size, any interim analysis would have too few events to be interpreted. Recruitment will be completed over 12–16 months, and given that the primary outcome has occurred by 30 days after the last patient is randomized, primary outcome assessment will have occurred by 18 months. Allowing for prolonged data collection for up to 3 months for secondary outcomes, the trial analyses will be completed less than 21 months after the trial commences.

## Discussion

The NEPTUNE trial is the first randomized controlled trial to investigate the role of iNPWT in reducing the incidence of SSIs in elective, open, CRR. Only one other study has been performed investigating the role of iNPWT following a CRR using a retrospective cohort design. Bonds et al., used iNPWT in patients following a CRR, at the request of the operating surgeon [17]. Although no difference was reported on univariate analysis, the use of iNPWT was found to be protective for SSI on multivariate analysis after controlling for various risk factors. Despite the positive findings and adjustments made, a significant selection bias exists in this study, and a randomized controlled trial is warranted. It is anticipated that the NEPTUNE trial will assist in the development of consensus recommendations for the use of iNPWT in patients following a CRR.

There are multiple theorized benefits of iNPWT in decreasing SSI rates. In a study by Wackenfors et al., laser Doppler velocimetry was used to assess the vascularity of incisions in a porcine model with NPWT. Increases in tissue microvascular perfusion were seen to correlate with the pressure applied in addition to increases in the partial pressure of oxygen within the soft tissue environment [18]. Other porcine models have demonstrated reduced rates of hematoma and serum formation in addition to increased lymphatic fluid clearance [19]. This is also associated with decreased shear forces and stresses as the negative pressure environment maintains tissue apposition. Incisional NPWT devices decrease the lateral stresses placed on incisions by approximately 50 %, leading to a distribution more consistent with that of intact tissue and further increases the force required to disrupt the incision [20]. The closed suction environment placed under sterile conditions also effectively separates the incision from the surrounding environment, thus theoretically preventing the inoculation of environmental bacteria.

This design of this trial will allow for strong conclusions to be made as to the role of iNPWT after CRR. The major limitation of this trial is in the inability to completely blind members of the clinical team, and the patient, to the treatment arm. Placebo control is not felt to be possible, as any additional dressing placed over the surgical wound may alter wound healing mechanics, and, therefore, likely impact on the rate of SSI. The idea of a “sham device” was initially entertained, but given the pragmatic nature of this trial, this or any alternate dressing would further fail to represent the current standard of care and use may result in misleading conclusions.

The results of this trial will be used to determine the role of iNPWT in the prevention of SSI after colorectal surgery. If this intervention is shown to be effective for the prevention of SSI in this high-risk population, significant benefit with respect to both patient morbidity and resource utilization may be achieved.

## Trial status

Enrollment started on 5 January 2015.

## Additional file

Additional file 1:
**Standard Protocol Items: Recommendations for Interventional Trials (SPIRIT) Checklist in word document (.doc) format.** The SPIRIT Checklist provides recommendations for the minimum set of scientific, ethical and administrative elements that should be addressed in a clinical trial protocol.

## Abbreviations

BMI: Body mass index
CDC: Center for Disease Control and Prevention
CRR: Colorectal resection
CTCAE: Common Terminology Criteria for Adverse Events
DSMB: Data Safety and Monitoring Board
ED: Emergency department
iNPWT: Incisional negative pressure wound therapy
KCI: Kinetic Concepts Inc.
LHSC: London Health Sciences Center
NPWT: Negative pressure wound therapy
PIMS: Prevena™ Incision Management System
SSI: Surgical site infection
